# Association of *Rahnella victoriana*, *Enterobacter hormaechei* subsp. *hoffmannii* and *Citrobacter braakii* with walnut decline

**DOI:** 10.1038/s41598-023-38427-9

**Published:** 2023-07-12

**Authors:** Mohammadreza Hajialigol, Nargues Falahi Charkhabi, Fatemeh Shahryari, Saadat Sarikhani

**Affiliations:** 1 Department of Entomology and Plant Pathology, College of Agricultural Technology, University College of Agriculture & Natural Resources, University of Tehran, Tehran, Iran; 2 Department of Plant Protection, Faculty of Agriculture, University of Zanjan, Zanjan, Iran; 3 Department of Horticulture, College of Agricultural Technology, University College of Agriculture & Natural Resources, University of Tehran, Tehran, Iran

**Keywords:** Microbiology, Plant sciences

## Abstract

Persian walnut (*Juglans regia*) has a considerable economic importance worldwide. However, the vigor and vitality of walnut trees were heavily affected by bark canker during the last few years. Irregular longitudinal cankers in the outer bark, stem tissue necrosis, and bleeding with black-colored exudates walnut trees were observed in Kermanshah, Hamedan, Markazi, Alborz, Isfahan, Qom, Semnan, and Razavi Khorasan provinces in western, central and eastern Iran during 2018 and 2019. A total of 150 symptomatic samples were collected from affected walnut trees in order to identify bacteria associated with walnut decline. Two-hundred sixty strains with a metallic green sheen were isolated on EMB-agar medium. The pathogenicity of all strains was proved by inoculating a suspension of the bacterial strains under the bark of immature walnut fruits cv. ‘Hartley’. Ninety-five strains caused necrosis and a dark-colored region in the mesocarp around the inoculation site 14 days post-inoculation. Moreover, 12 representative strains induced necrotic and black-colored tissues in the bark of young green twigs of two-year old walnut seedling cv. ‘Chandler’. The strains were classified into four categories based on conventional phenotypic characters confirmed with the 16S rRNA gene sequences. A phylogenetic tree based on the concatenated sequences of two housekeeping gene fragments, *gyrB* and *infB*, indicated that strains including I1, Q6, and S6 were grouped in a cluster with *Gibbsiella quercinecans* FBR97^T^ as well as strains I2, I5, and KE6 were clustered with *Rahnella victoriana* FRB 225^T^. Moreover, strains MR1, MR3, and MR5 were grouped with the *Enterobacter hormaechei* subsp. *hoffmannii* DSM 14563^T^. The phylogenetic analyses based on the partial sequencing of housekeeping genes including *fusA*, *pyrG*, and *leuS* revealed that strains KH1, KH3, and KH7 belong to *Citrobacter braakii* species. To the best of our knowledge, this is the first report of *C. braakii* and *E. hormaechei* as plant pathogens and *R. victoriana* associated with walnut decline.

## Introduction

Persian walnut (*Juglans regia*) considered as a strategic crop with high economic and nutritional value worldwide due to positive effects on human health and timber industry. Iran, with 356,666 tons of nuts in the shell, ranks three in walnut production after the United States of America and China^1^. However, the health and vitality of walnut trees were heavily affected by bark canker during the last few years^2,3^.

Walnut canker is characterized by brown to blackish-brown round blotches on the trunks and main branches, necrosis of inner bark and bleeding with dark brown to black-colored exudates. This disease may result in progressive decline, loss in vigor over time and occasionally complete tree death^3–5^. Shallow and deep bark canker of walnut caused by *Brenneria nigrifluens* and *Brenneria rubrifaciens* respectively, were reported from different countries, including the USA^4,5^, Spain^6,7^, Italy^8^, France^9^, and Turkey^10^. Walnut canker caused by *B. nigrifluens* and *B. rubrifaciens* was recorded in multiple regions of Iran^2,11–13^. Furthermore, the number of the bacteria identified as causative agents of walnut canker was increased during the last few years. *Brenneria rosae* subsp. *rosae* and *Gibbsiella quercinecans* were recently recognized as responsible for walnut stem bleeding and decline in northwestern Iran^3,14^.

Sequencing and analysis of the 16S rRNA gene has limitations in determining relationships at the species level because of low discrimination power^15^. Therefore, the multilocus sequence analysis (MLSA) approach is used for the phylogenetic analyses. Multiple housekeeping genes, such as *gyrB*, *infB*, *rpoD*, and *rpoB*^16–18^, *dnaJ*^19^ and *recA*^20^ is established reliability in species delineation and strain identification in Enterobacterales. Moreover, phylogenetic relationships among *Citrobacter* species are characterized by sequencing internal portions of genes *rpoB*, *fusA*, *pyrG*, *leuS*, and 16S rRNA^21^.

Walnut decline symptoms were observed on walnut trees in multiple provinces of Iran in 2018 and 2019. The present study aimed to identify the causative agents of walnut decline by their phenotypic features, approval of pathogenicity, the partial sequencing of the 16S rRNA gene and MLSA.

## Results

### Symptoms and bacterial isolation

**Figure 1:**
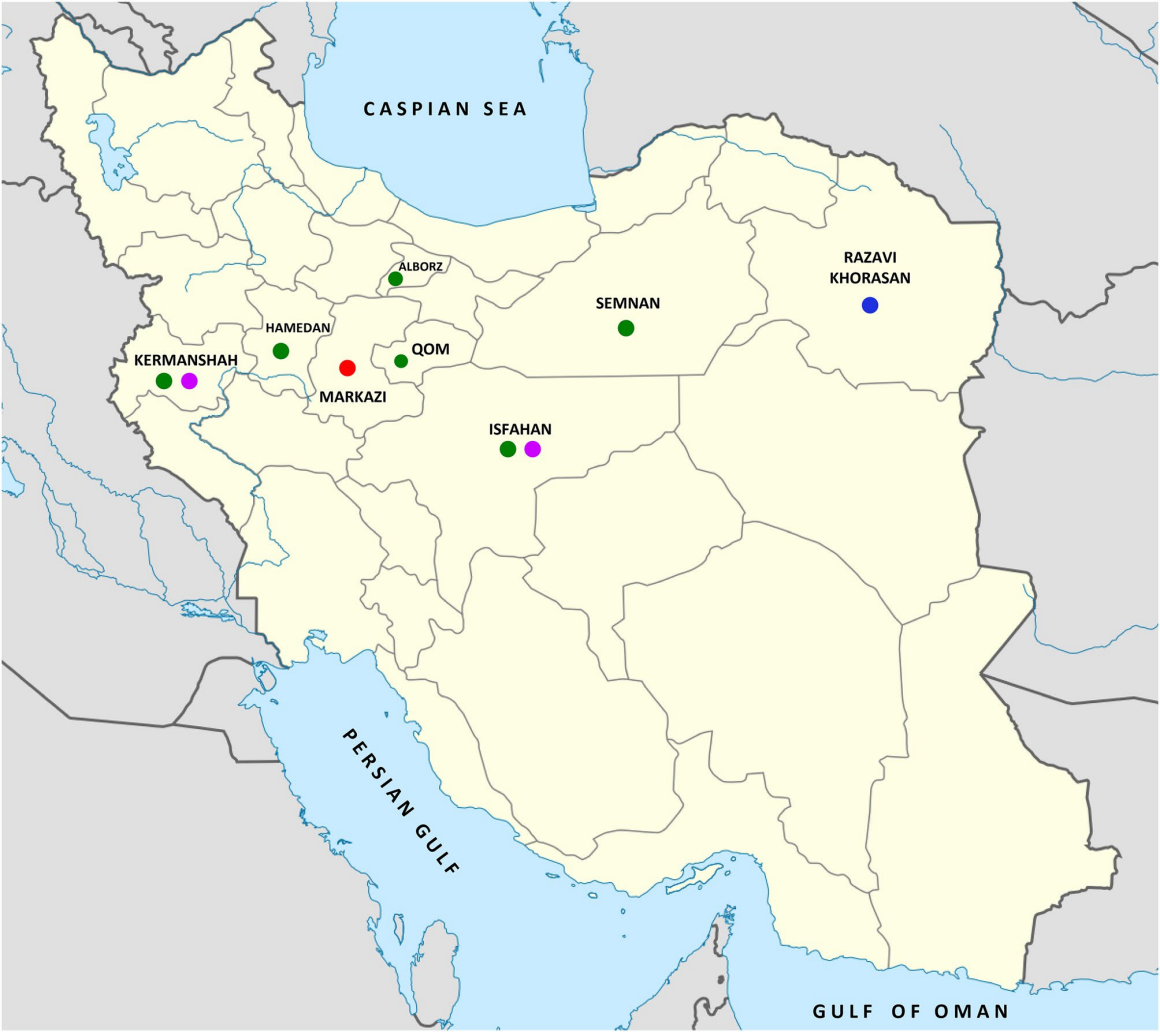
Map showing the sampling regions and bacterial species recognized in association with walnut decline in each province. Colored rings represent the bacterial species: *Gibbsiella quercinecans* (green), *Rahnella victoriana* (purple); *Enterobacter hormaechei* subsp. *hoffmannii* (red), and *Citrobacter braakii* (blue). Vector map were downloaded from https://commons.wikimedia.org/wiki/File:Iran_location_map.svg and edited using Microsoft PowerPoint 2010.

**Figure 2:**
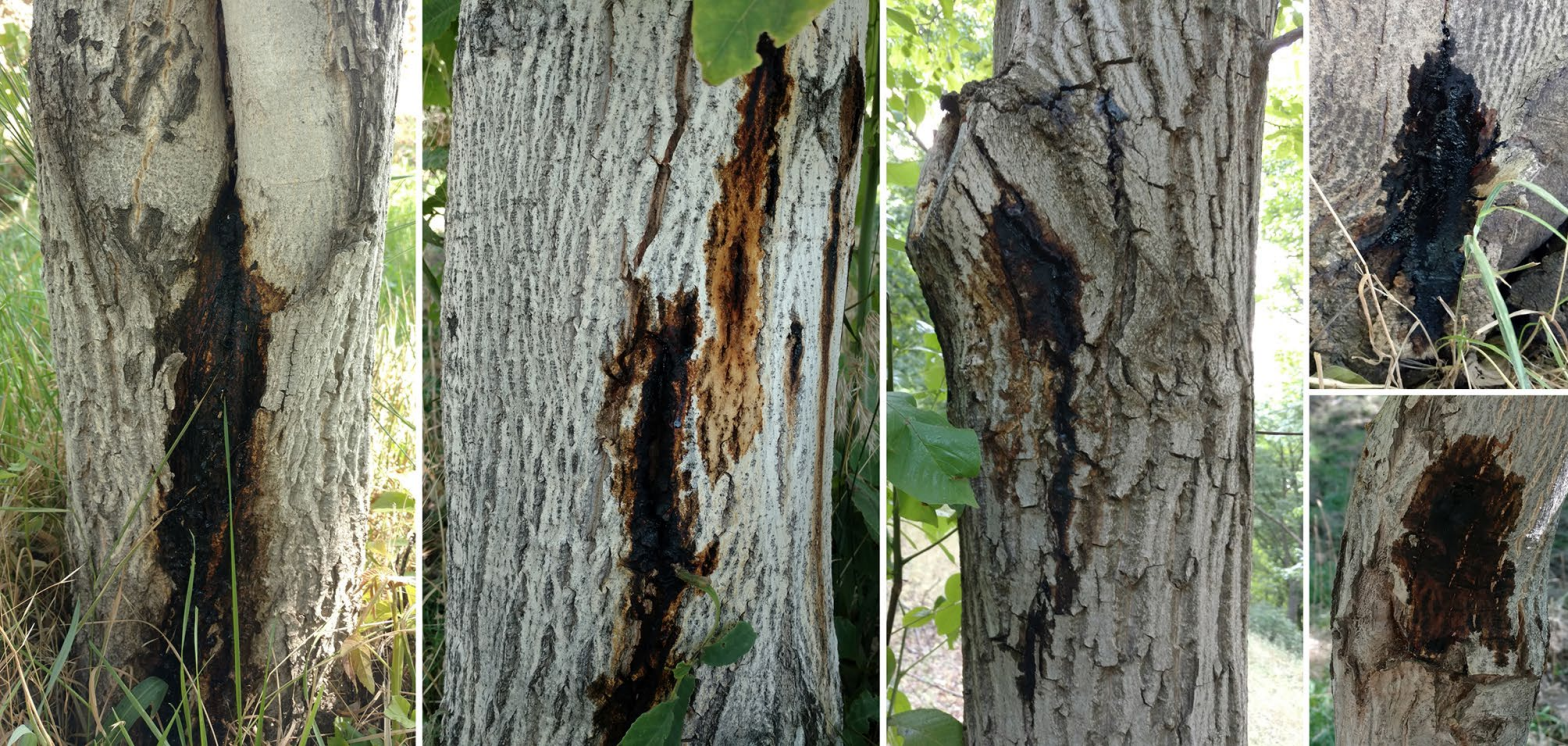
Symptoms of walnut decline in Iran, canker lesions, stem bleeding of a dark sticky fluid from vertical cracks.

A total of 150 symptomatic samples were collected from affected walnut trees of Kermanshah, Hamedan, Markazi, Alborz, Isfahan, Qom, Semnan, and Razavi Khorasan, provinces in western, central and eastern Iran (Fig. 1). The frequent symptoms were irregular longitudinal cankers in the outer bark, stem tissue necrosis, bleeding with black-colored exudates, and dark lesions in the inner bark (Fig. 2). Walnut trees seriously affected by cankers showed a progressive loss in vigor and death after few years. The severity of the disease symptoms was higher in some areas, especially in Kermanshah, Hamedan, and Qom provinces. Two-hundred sixty strains with metallic green sheen were isolated on eosin methylene blue agar (EMB-agar) medium. The colony of bacterial strains on nutrient agar (NA) was cream, round, convex and smooth with entire margins.

### Pathogenicity assay

**Figure 3:**
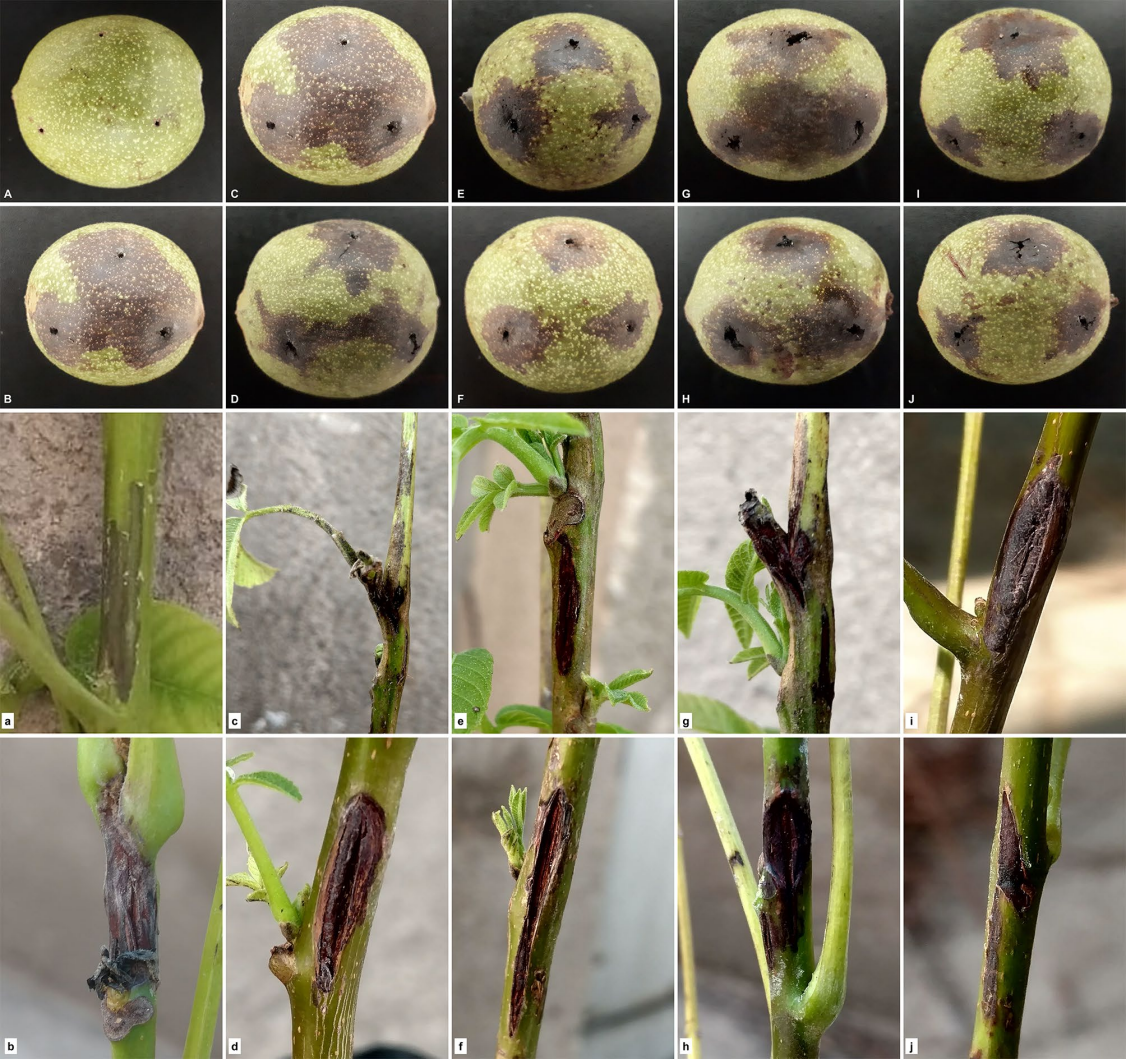
Pathogenicity assays of strains isolated from walnut canker symptoms from different provinces of Iran on immature fruit and seedlings, 14 and 30 days after inoculation, respectively. (**A**, **a**) Negative control infiltrated using sterile deionized water, immature fruit and seedlings inoculated using strains *Brenneria nigrifluens* M2 (**B**, **b**) as positive control; *Gibbsiella quercinecans* strains I1 (**C**, **c**) and Q6 (**D**, **d**); *Rahnella victoriana* strains I2 (**E**, **e**) and I5 (**F**, **f**); *Enterobacter hormaechei* subsp. *hoffmannii* strains MR1 (**G**, **g**) and MR7 (**H**, **h**); and *Citrobacter braakii* strains KH1 (**I**, **i**) and KH3 (**J**, **j**).

The pathogenicity of 95 strains was proved on immature walnut fruits cv. ‘Hartley’. Necrosis and a dark-colored region in the mesocarp around the inoculation site were observed 14 days post-inoculation (Table 1). The pathogenicity of 12 representative strains was confirmed on young green twigs of two-year-old walnut seedling cv. ‘Chandler’. Necrotic and black-colored tissues appeared in the bark around the inoculation points, and such symptoms were detected in positive control, *B. nigrifluens* M2, 30 days post-inoculation (Fig. 3). *G. quercinecans* and *Enterobacter hormaechei* subsp. *hoffmannii* strains induced large necrotic areas beyond inoculated sites on immature fruits and branches (Fig. 3). No symptoms were detected in the fruits and young branches infiltrated with sterile distilled water (SDW). Bacteria resembled those used in inoculations were recovered in high populations from the inoculated immature fruits and twigs 14 and 30 days after inoculation, respectively. Re-isolated strains were identical with inoculated ones in all phenotypic characters.

**Table 1:** Code, bacterial species, the origin and sampling date of pathogenic bacterial strains isolated from walnut (*Juglans regia*) with decline symptoms from Iran.

Code	Species	Origin	Sampling date
A1	*Gibbsiella quercinecans*	Alborz, Karaj	September 2018
A3	*G. quercinecans*	Alborz, Karaj	September 2018
A5	*G. quercinecans*	Alborz, Karaj	September 2018
A8	*G. quercinecans*	Alborz, Karaj	September 2018
A10	*G. quercinecans*	Alborz, Karaj	September 2018
A11	*G. quercinecans*	Alborz, Karaj	September 2018
A12	*G. quercinecans*	Alborz, Karaj	September 2018
A15	*G. quercinecans*	Alborz, Karaj	September 2018
A18	*G. quercinecans*	Alborz, Karaj	September 2018
A22	*G. quercinecans*	Alborz, Karaj	September 2018
A27	*G. quercinecans*	Alborz, Karaj	September 2018
A29	*G. quercinecans*	Alborz, Karaj	September 2018
A30	*G. quercinecans*	Alborz, Karaj	September 2018
I1	*G. quercinecans*	Isfahan, Khansar	August 2018
I6	*G. quercinecans*	Isfahan, Khansar	August 2018
I7	*G. quercinecans*	Isfahan, Khansar	August 2018
I9	*G. quercinecans*	Isfahan, Khansar	August 2018
I11	*G. quercinecans*	Isfahan, Khansar	August 2018
I16	*G. quercinecans*	Isfahan, Khansar	August 2018
I15	*G. quercinecans*	Isfahan, Khansar	August 2018
H1	*G. quercinecans*	Hamadan, Tuyserkan	August 2018
H3	*G. quercinecans*	Hamadan, Tuyserkan	August 2018
H4	*G. quercinecans*	Hamadan, Tuyserkan	August 2018
H6	*G. quercinecans*	Hamadan, Tuyserkan	August 2018
H7	*G. quercinecans*	Hamadan, Tuyserkan	August 2018
H11	*G. quercinecans*	Hamadan, Tuyserkan	August 2018
H12	*G. quercinecans*	Hamadan, Tuyserkan	August 2018
H13	*G. quercinecans*	Hamadan, Tuyserkan	August 2018
H14	*G. quercinecans*	Hamadan, Tuyserkan	August 2018
H15	*G. quercinecans*	Hamadan, Tuyserkan	August 2018
H18	*G. quercinecans*	Hamadan, Tuyserkan	August 2018
H19	*G. quercinecans*	Hamadan, Tuyserkan	August 2018
H23	*G. quercinecans*	Hamadan, Tuyserkan	August 2018
H25	*G. quercinecans*	Hamadan, Tuyserkan	August 2018
H21	*G. quercinecans*	Hamadan, Tuyserkan	August 2018
KE1	*G. quercinecans*	Kermanshah, Sahneh	August 2018
KE2	*G. quercinecans*	Kermanshah, Sahneh	August 2018
KE5	*G. quercinecans*	Kermanshah, Sahneh	August 2018
KE6	*G. quercinecans*	Kermanshah, Sahneh	August 2018
KE8	*G. quercinecans*	Kermanshah, Sahneh	August 2018
KE12	*G. quercinecans*	Kermanshah, Sahneh	August 2018
KE17	*G. quercinecans*	Kermanshah, Sahneh	August 2018
KE18	*G. quercinecans*	Kermanshah, Paveh	September 2018
KE19	*G. quercinecans*	Kermanshah, Paveh	September 2018
KE22	*G. quercinecans*	Kermanshah, Paveh	September 2018
KE26	*G. quercinecans*	Kermanshah, Paveh	September 2018
Q5	*G. quercinecans*	Qom, Dastjerd	August 2018
Q6	*G. quercinecans*	Qom, Dastjerd	August 2018
Q9	*G. quercinecans*	Qom, Dastjerd	August 2018
Q10	*G. quercinecans*	Qom, Dastjerd	August 2018
Q11	*G. quercinecans*	Qom, Dastjerd	August 2018
Q14	*G. quercinecans*	Qom, Dastjerd	August 2018
Q16	*G. quercinecans*	Qom, Dastjerd	August 2018
Q18	*G. quercinecans*	Qom, Dastjerd	August 2018
S2	*G. quercinecans*	Semnan, Shahmirzad	September 2018
S3	*G. quercinecans*	Semnan, Shahmirzad	September 2018
S4	*G. quercinecans*	Semnan, Shahmirzad	September 2018
S5	*G. quercinecans*	Semnan, Shahmirzad	September 2018
S6	*G. quercinecans*	Semnan, Shahmirzad	September 2018
S14	*G. quercinecans*	Semnan, Shahmirzad	September 2018
S15	*G. quercinecans*	Semnan, Shahmirzad	September 2018
S39	*G. quercinecans*	Semnan, Shahmirzad	September 2018
I2	*Rahnella victoriana*	Isfahan, Khansar	August 2018
I3	*R. victoriana*	Isfahan, Khansar	August 2018
I4	*R. victoriana*	Isfahan, Khansar	August 2018
I5	*R. victoriana*	Isfahan, Khansar	August 2018
I8	*R. victoriana*	Isfahan, Khansar	August 2018
I13	*R. victoriana*	Isfahan, Khansar	August 2018
I14	*R. victoriana*	Isfahan, Khansar	August 2018
I18	*R. victoriana*	Isfahan, Khansar	August 2018
I20	*R. victoriana*	Isfahan, Khansar	August 2018
KE10	*R. victoriana*	Kermanshah, Paveh	September 2018
KE7	*R. victoriana*	Kermanshah, Paveh	September 2018
KE16	*R. victoriana*	Kermanshah, Paveh	September 2018
KE18	*R. victoriana*	Kermanshah, Paveh	September 2018
KE20	*R. victoriana*	Kermanshah, Paveh	September 2018
MR1	*Enterobacter hormaechei* subsp. *hoffmannii*	Markazi, Mahallat	August 2018
MR2	*E. hormaechei* subsp. *hoffmannii*	Markazi, Mahallat	August 2018
MR3	*E. hormaechei* subsp. *hoffmannii*	Markazi, Mahallat	August 2018
MR4	*E. hormaechei* subsp. *hoffmannii*	Markazi, Mahallat	August 2018
MR5	*E. hormaechei* subsp. *hoffmannii*	Markazi, Mahallat	August 2018
MR6	*E. hormaechei* subsp. *hoffmannii*	Markazi, Mahallat	August 2018
MR7	*E. hormaechei* subsp. *hoffmannii*	Markazi, Mahallat	August 2018
MR8	*E. hormaechei* subsp. *hoffmannii*	Markazi, Mahallat	August 2018
MR9	*E. hormaechei* subsp. *hoffmannii*	Markazi, Mahallat	August 2018
MR10	*E. hormaechei* subsp. *hoffmannii*	Markazi, Mahallat	August 2018
MR11	*E. hormaechei* subsp. *hoffmannii*	Markazi, Mahallat	August 2018
KH1	*Citrobacter braakii*	Razavi Khorasan, Bardeskan	July 2019
KH2	*C. braakii*	Razavi Khorasan, Bardeskan	July 2019
KH3	*C. braakii*	Razavi Khorasan, Bardeskan	July 2019
KH4	*C. braakii*	Razavi Khorasan, Bardeskan	July 2019
KH5	*C. braakii*	Razavi Khorasan, Bardeskan	July 2019
KH6	*C. braakii*	Razavi Khorasan, Bardeskan	July 2019
KH7	*C. braakii*	Razavi Khorasan, Bardeskan	July 2019
KH8	*C. braakii*	Razavi Khorasan, Bardeskan	July 2019

### Phenotypic characteristics

All strains were negative in Gram reaction, oxidase test and levan sucrase activity. All strains were fermentative aerobes and catalase positive. None of strains produced pink or fluorescent pigments on YDC and King’s B media, respectively. The strains were classified into four categories based on conventional phenotypic characters. Sixty-two strains belonging to *G. quercinecans* were negative for gelatin and starch hydrolysis, whereas positive for indole production and esculin hydrolysis. Fourteen strains characterized as *Rahnella victoriana* were able to hydrolyze Tween 20, Tween 80, gelatin and esculin. *R. victoriana* strains did not produce indole or urease. The third phenotypic group consists of 11 strains was identified as *Enterobacter hormaechei* subsp*. hoffmannii*, which hydrolyzed gelatin, however, did not utilize starch, esculin, Tween 20 and Tween 80. These strains produced indole and urease (Table 2). The last phenotypic group, including eight strains was recognized as* Citrobacter braakii*, which utilize starch. Strains of this group did not utilized esculin, Tween 20, Tween 80 and gelatin (Table 2).

**Table 2:** The accession numbers of 16S rRNA and housekeeping genes sequences deposited in GenBank.

Species	Strain	16S rRNA	*gyrB*		*infB*
*Gibbsiella quercinecans*	I1	OP048975	OP057241		OP057250
	Q6	–	OP057242		OP057251
	S6	–	OP057243		OP057252
*Rahnella victoriana*	I2	OP048977	OP057247		OP057256
	I5	–	OP057248		OP057257
	KE7	–	OP057249		OP057258
*Enterobacter hormaechei* subsp. *hoffmannii*	MR1	OP048976	OP057244		OP057253
	MR3	–	OP057245		OP057254
	MR5	–	OP057246		OP057255
		16S rRNA	*fusA*	*leuS*	*pyrG*
*Citrobacter braakii*	KH1	–	OP057224	OP057227	OP057230
	KH3	–	OP057225	OP057228	OP057231
	KH7	OP048974	OP057226	OP057229	OP057232

### Phylogenetic analysis

Amplification and partial sequencing of 16S rRNA gene of one representative strain from each phenotypic group were performed. Moreover, the *gyrB* and *infB* of nine representative strains of three phenotypic groups and *fusA*, *pyrG*, and *leuS* of those of fourth group were amplified and partially sequenced. The accession numbers of sequences obtained in this study were deposited in the NCBI GenBank database are listed in Table 3.

**Table 3:** Phenotypic characters of walnut pathogenic strains isolated from decline symptoms from different provinces of Iran.

Characteristic	*Gibbsiella quercinecans* (62 strains)	*Gibbsiella quercinecans *FBR97^T^	*Rahnella victoriana *(14 strains)	*Rahnella victoriana* FBR225^T^	*Enterobacter hormaechei* subsp*. hoffmannii *(11 strains)	*Citrobacter braakii *(8 strains)
Host	Walnut	Oak	Walnut	Oak	Walnut	Walnut
Gram	−	−	−	−	−	−
Oxidative/fermentative growth	Fermentative	Fermentative	Fermentative	Fermentative	Fermentative	Fermentative
Catalase	+	+	+	+	+	+
Oxidase	−	−	−	−	−	−
Indole production	+	+	−	−	+	+
Urease	−	−	−	−	+	+
Hydrolysis of starch	−	−	−	−	−	+
Hydrolysis of esculin	+	+	+	+	−	−
Hydrolysis of gelatin	−	−	+	+	+	−
Levan	−	−	−	−	−	−
Tween 20	−	−	+	+	−	−
Tween 80	−	−	+	+	−	−
Pigmentation on YDC	−	−	−	−	−	−
Pigmentation on King’s B	−	−	−	−	−	−
5% salt tolerance	+	+	+	+	+	+
l-Proline	−	−	−	−	+	−
l-Alanine	−	−	−	−	−	−
l-Histidine	+	+	+	+	+	+
l-Cysteine	−	−	−	−	−	−
l-Ornithine	−	−	−	−	−	+
l-Lysine	−	−	−	−	−	−
l-Tryptophan	+	+	+	+	+	+
l-Arginine	−	−	−	−	−	−
l-Serin	−	−	−	−	−	−
Maltose	+	+	+	+	+	+
d-Trehalose	+	+	+	+	+	+
d-Lactose	+	+	+	+	+	+
l-Arabinose	+	+	+	+	+	+
d-Sorbitol	+	+	+	+	−	+
d-Mannitol	+	+	+	+	+	+
d-Fructose	+	+	+	+	+	+
Sucrose	+	+	−	−	−	−
Glycerol	+	+	+	+	+	+

*Gibbsiella quercinecans* FBR97^T^ and *Rahnella victoriana* FBR225^T^ strains used as control.

**Figure 4:**
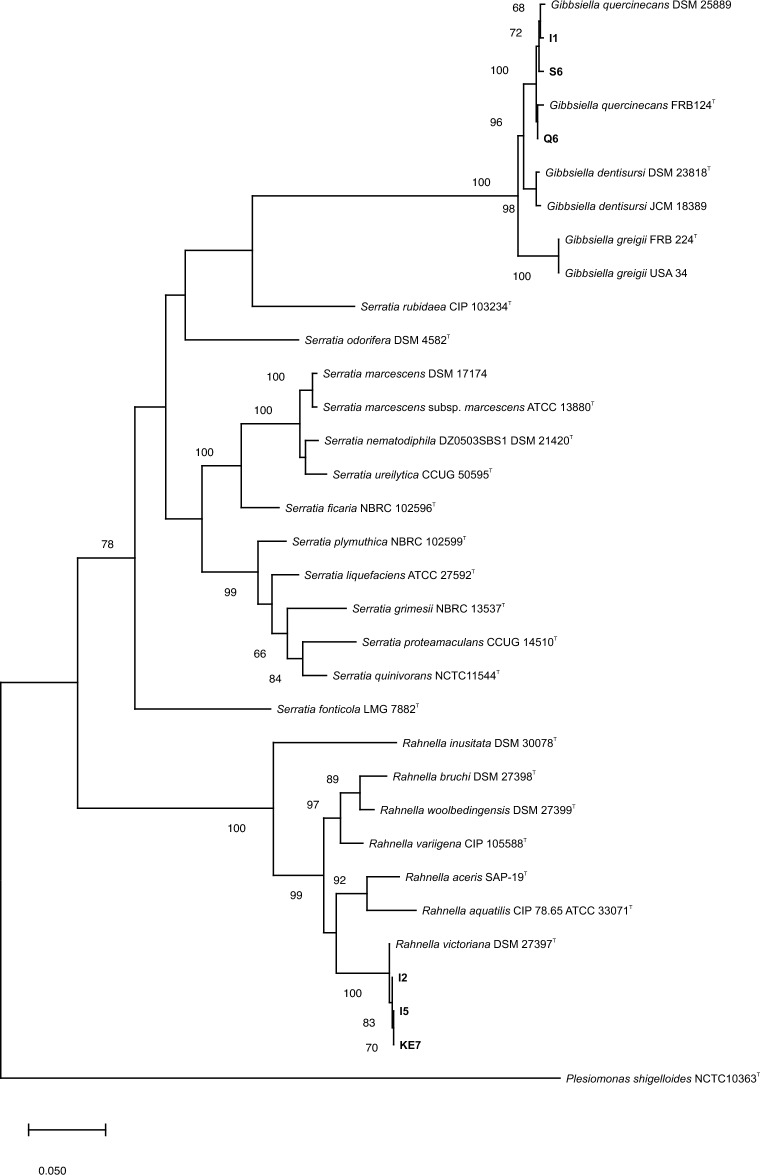
Maximum likelihood phylogenetic tree based on based on concatenated sequences of *gyrB* and *infB* genes showing the taxonomic position of *Gibbsiella quercinecans* (I1, S6 and Q6) and *Rahnella victoriana* (I2, I5 and KE7) strains isolated from walnut decline symptoms. The tree was constructed using a general time reversible substitution model with a gamma distribution and invariant sites (GTR + G + I) based on the lowest Bayesian information criterion (BIC) score. Bootstrap values calculated for 1000 replications are indicated. *Plesiomonas shigelloides* NCTC10363^T^ used as outgroup.

Pairwise comparison the 16S rRNA gene nucleotide sequences of strains I1 and I2 were 100% identical with those of *G. quercinecans* FBR97^T^ and *R. victoriana* FRB 225^T^, respectively. Moreover, a phylogenetic tree reconstructed based on the concatenated sequences of two housekeeping gene fragments, *gyrB* (601 bp) and *infB* (615 bp), revealed that the strains I1, Q6 and S6 were grouped in a cluster with *G. quercinecans* FBR97^T^. Additionally, the strains I2, I5, and KE6 were clustered with *R. victoriana* FRB 225^T^, not only in the phylogenetic tree based on the concatenated sequences (Fig. 4), but also in each of the single gene-based trees (Data not shown).

**Figure 5:**
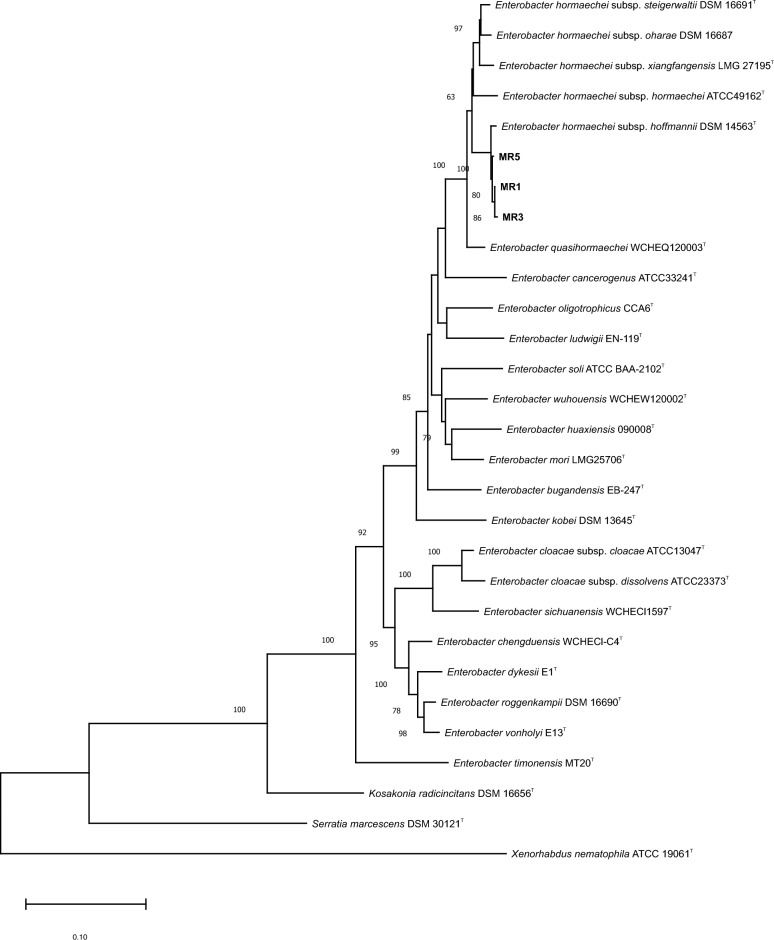
Maximum likelihood phylogenetic tree based on based on concatenated sequences of *gyrB* and *infB* genes showing the taxonomic position of *Enterobacter hormaechei* subsp. *hoffmannii* strains (MR1, MR3 and MR5) isolated from walnut decline symptoms. The tree was constructed using a general time reversible substitution model with a gamma distribution and invariant sites (GTR + G + I) based on the lowest Bayesian information criterion (BIC) score. Bootstrap values calculated for 1000 replications are indicated. *Xenorhabdus nematophila* ATCC 19061^T^ (DSM 3370^T^) used as outgroup.

The partial 16S rRNA gene sequence of strain MR1 was identical to that of *E. hormaechei* subsp. *hoffmannii* DSM 14563^T^. The maximum-likelihood phylogenic tree, including strains identified as *Enterobacter* were collected from Markazi province and type strains of *Enterobacter* species was created using concatenated sequences of *gyrB* (684 bp) and *infB* (906 bp) genes. The strains MR1, MR3, and MR5 were grouped with *E. hormaechei* subsp. *hoffmannii* DSM 14563^T^. The resulting cluster presented a robust bootstrap value (Fig. 5).

**Figure 6:**
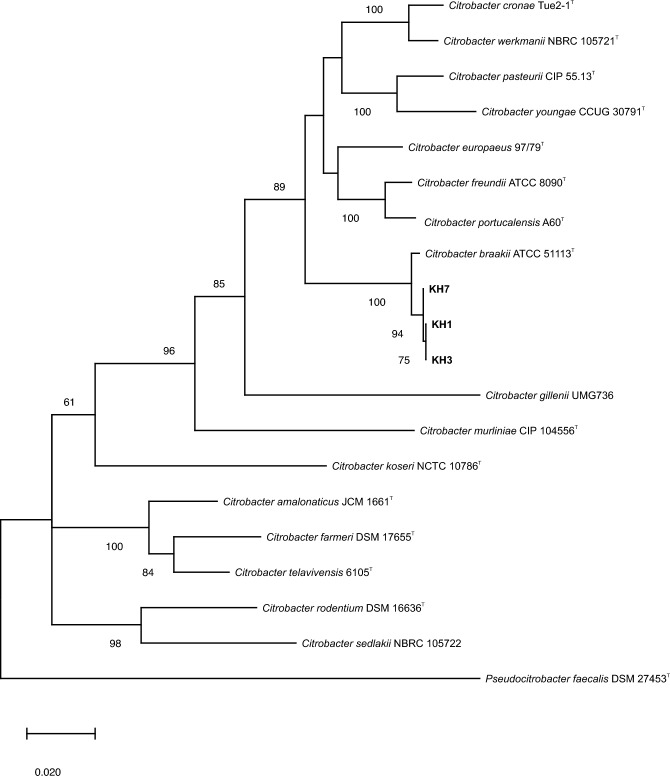
Maximum likelihood phylogenetic tree based on based on concatenated sequences of *fusA*, *pyrG*, and *leuS* genes showing the taxonomic position of *Citrobacter braakii* strains (KH1, KH3 and KH7) isolated from walnut decline symptoms. The tree was constructed using a general time reversible substitution model with a Tamura-Nei model with a gamma distribution and invariant sites (TN93 + G + I) based on the lowest Bayesian information criterion (BIC) score. Bootstrap values calculated for 1000 replications are indicated. *Pseudocitrobacter faecalis* DSM 27453^T^ was used as outgroup.

The partial 16S rRNA gene sequence of strain KH7 collected from Razavi Khorasan province indicated that this pathogenic strain on walnut belongs to *Citrobacter* genus with 99.63 and 99.44% similarity with *Citrbacter freundii* ATCC 8090^T^ and* Citrobacter braakii* ATCC5113^T^, respectively. The phylogenetic analyses based on the partial sequencing of three housekeeping genes, *fusA* (633 bp), *pyrG* (305), and *leuS* (640 bp) demonstrated that strains KH1, KH3, and KH7 belong to *C. braakii* species in a monophyletic clade with high bootstrap support (Fig. 6).

As Fig. 1 shows, *G. quercinecans* was identified associated with walnut decline in all sampled provinces except Markazi and Razavi Khorasan whereas the agents of disease in theses provinces were *E. hormaechei* subsp. *hoffmannii* and *C. braakii*, respectively. Furthermore, *R. victoriana* strains were in association with walnut decline symptoms from Isfahan and Kermanshah provinces.

## Discussion

Iran is the third producer of Persian walnut worldwide. However, its walnut trees have been under threat from decline during last decade. The causal agents of disease were identified in the southern, northern, and northwestern provinces^2,12–14^. Nevertheless, despite high severity and prevalence of walnut decline, the etiology of the disease has not yet been studied in the most western, central and eastern provinces. In this study, the bacterial strains in association with the walnut decline samples originating from eight provinces of Iran were identified using classical and molecular methods. The pathogenic bacterial strains were categorized into four species including *G. quercinecans*, *R. victoriana*, *C. braakii*, and *E. hormaechei* subsp. *hoffmannii*.

*G. quercinecans* was recognized as the causal agent of acute oak decline (AOD) in the United Kingdom^22^, Spain^23,24^, Switzerland^25^, Iran^26^ and Poland^27^. In addition, it was known as the causal agents of the necrotic tissues of apple and pear trees in Germany and Austria, as well as exudates from a pear tree in China^28^. Furthermore, *G. quercinecans* was reported as the causal agents of the bacterial canker disease of Russian olive^29^, and the walnut canker disease in Iran^3^. *R. victoriana* was isolated from oak trees suffering AOD in the UK^18^ and Iran^30^. Moreover, *R. victoriana* strain JZ-GX1 was identified as a growth-promoting rhizobacteria on poplar roots^31^. To the best of our knowledge, this is the first report of *R. victoriana* in association with walnut decline.

Pathogenicity of *G. quercinecans* strains is attributed to production of plant cell wall-degrading enzymes (PCWDEs) such as rhamnogalacturonase, cellulase, β-glucosidase, α-galactosidase, galactanase, and polyglactoronase whereas pathogenicity of *R. victoriana* species is ascribed to α- and β-galactosidase, cellulose and a type III secretion system^32^. *R. victoriana* and *G. quercinecans* were isolated from symptomatic walnut trees in Isfahan and Kermanshah provinces. Nevertheless, the role of individual lesion microbiota as components of a complex pathobiome, is unknown.

Human bacterial pathogens which cause diseases in plants and vice versa are dramatically increased during the last decades. For instance, *Burkholderia gladioli* and *Burkholderia glumae*, routinely isolated from panicle blight of rice^33^, were identified in pneumonia infections in patients with the chronic granulomatous disease^34,35^. *Agrobacterium pusense* inciting sepsis in human beings^36^, induced tumors on Lawson cypress crown^37^. Moreover, *Erwinia billingiae*, causing mango bacterial canker disease^38^, was known as the agent of cutaneous infection and bacteremia in humans^39^. *Stenotrophomonas maltophilia*, an emerging human pathogen^40^, is also responsible for necrotic lesions and stem bleeding on oak trees in Iran^41^. In addition, *C. freundii,* a frequent cause of nosocomial infections, was recognized as the agent of canker on white mulberry trunks and scaffold branches of trees^42,43^. Species *C. braakii* considered an as opportunistic human pathogen, was in association with various infections^44^. Furthermore, *E. hormaechei* is a causative pathogen for human nosocomial infections^45^. These data reveal that *C. braakii* and *E. hormaechei* subsp. *hoffmannii* are new plant pathogens which cause walnut decline.

Overall, the present study highlights the fact that walnut decline has to be considered a complex disease in which several bacteria are involved. Identification and characterization of all bacteria associated with walnut decline will eventually improve our understanding of the etiology of the disease and may result in improved management techniques for control. Future research is necessary to find beneficial bacteria serves as biological control for a variety of bacterial agent of walnut decline or finding resistant genotypes.

## Material and methods

### Sampling and bacterial isolation

Symptomatic samples were collected from walnut orchards of different provinces of Iran including, Kermanshah, Hamedan, Markazi, Alborz, Isfahan, Qom, Semnan, and Razavi Khorasan during 2018–2019. The samples were washed under tap water for 5 min and were disinfected using ethanol 70% for 30 s and were rinsed three times with SDW. Then the symptomatic tissues were grounded in SDW and incubated at room temperature for one hour. Additionally, more pieces were incubated in 20 ml SDW in incubator shaker for 24 h, and a loopful of resulting suspensions were streaked on plates of EMB-agar (Merck, Darmstadt, Germany) medium containing 1% glycerol and 0.5% yeast extract and were incubated at 28 °C for 72 h. Single colonies were purified on NA plates and routinely maintained in 15% glycerol stocks at − 80 °C.

### Pathogenicity assays

Pathogenicity of all isolated strains was carried out on walnut immature fruits cv. ‘Hartley’. The fruits were disinfected using ethanol 70% for three min^46^. Twenty µl of bacterial suspension (10^6^ CFU/ml) were infiltrated in each inoculation site. Inoculated fruits were incubated in Ziploc bags at 26 °C with 90% relative humidity^47^. Each strain was inoculated in three replicates. Symptom progression was observed for 14 days. Moreover pathogenicity of 12 representative strains (namely I1, Q6, S6, I2, I5, KE6, MR1, MR3, MR5, KH1, KH3, and KH7) was performed by inoculating 60 μl of bacterial suspension (10^8^ CFU/ml) on green twigs of two-years old walnut clonal seedlings cv. Chandler. The inoculated stems were covered using parafilm and maintained in a greenhouse under a cycle of 16 h light at 28 °C and 8 h dark at 24 °C with 75% relative humidity. Symptoms were monitored one-month post-inoculation. *B. nigrifluens* M2^13^ and SDW were used as positive and negative controls, respectively. Pathogenicity assays were repeated at least twice. To fulfill Koch's postulates, re-isolation from 12 inoculated fruits and branches was performed 14- and 30- days post-inoculation on EMB-agar, respectively. The re-isolated strains were identified by phenotypic characters.

### Phenotypic characteristic

All pathogenic strains as well as 12 re-isolated strains from walnut inoculated immature fruits and twigs namely I1, Q6, S6, I2, I5, KE6, MR1, MR3, MR5, KH1, KH3, and KH7 were subjected to physiological, morphological and biochemical tests including, Gram^48^, oxidase reactions^49^, fermentative metabolism^50^, and levan formation from sucrose. Moreover, the production of pink and fluorescent pigments on YDC and King’s B was performed^51^. Hydrolysis of starch, esculin, Tween 20, and Tween 80 was performed according to Schaad et al.^51^. Utilization of carbon sources was assayed using the basal medium of Ayers et al.^52^ complemented with filter-sterilized carbohydrate sources at 0.25% final concentration. *G. quercinecans* LMG25500^T^ and *R. victoriana* FRB 225^T^ were also used as reference strains in all tests.

### Phylogenetic analysis

Genomic DNA was extracted from 24 h culture using the alkaline lysis^53^. The PCR was performed in a volume of 30 µl containing 2 × *Taq* DNA Polymerase MasterMix (Ampliqon A/S, Odense, Denmark), 10 pmol of each primer, and 1 µl of template DNA. PCR was performed in BioER XP cycler (Zhejiang, China).

The PCR amplification of the 16S rRNA gene of four representative strains including I1, I2, MR1, and KH7 was performed using the primer pair 27F/1492R^54,55^. PCR was performed under the following conditions initial denaturation 5 min at 94 °C followed by 30 cycles of 35 s at 94 °C, 30 s at 55 °C and 2 min at 72 °C, with a final extension of 10 min at 72 °C.

Amplification of *gyrB* and *infB* genes was performed using gyrB 01-F/gyrB 02-R and infB 01-F/infB 02-R primer pairs (Table 4^16^), respectively using the conditions previously published^16^.

**Table 4:** Name, sequence, amplicon size and reference of primers used in this study.

Name	Sequences (5′–3′)	Amplicon size (bp)	Reference
27F	AGA GTT TGA TCC TGG CTC AG	1500	^54^
1492R	TAC GGC TAC CTT GTT ACG ACTT		^55^
*gyrB* 01-F	TAA RTT YGA YGA YAA CTC YTA YAA AGT	971	^16^
*gyrB* 02-R	CMC CYT CCA CCA RGT AMA GTT		
gyrB 07-F	GTV CGT TTC TGG CCV AG	Sequencing primer	^16^
*infB* 01-F	ATY ATG GGH CAY GTH GAY CA	1124	^16^
*infB* 02-R	ACK GAG TAR TAA CGC AGA TCC A		
infB 03-F	ACG GBA TGA TYA CST TCC TGG	Sequencing primer	^16^
fusA3	CAT CGG TAT CAG TGC KCA CAT CGA	801	^56^
fusA4	CAG CAT CGC CTG AAC RCC TTT GTT		
leuS3	CAG ACC GTG CTG GCC AAC GAR CAR GT	806	^56^
leuS4	CGG CGC GCC CCA RTA RCG CT		
pyrG3	GGG GTC GTA TCC TCT CTG GGT AAA GG	429	^56^
pyrG4	GGA ACG GCA GGG ATT CGA TAT CNC CKA		

The PCR program used for amplification of *fusA*, *leuS*, and *pyrG* genes consisted of an initial denaturation 2 min at 94 °C followed by 10 cycles of 1 min at 94 °C, 1 min at 60 °C and 1 min at 72 °C, in the following, 20 cycles of 1 min at 94 °C, 1 min at 60 °C and 1 min at 72 °C with a final extension of 5 min at 72 °C (Table 4^56^). The PCR products were electrophoresed in 0.5 × TBE buffer (tris–borate) and visualized by Safe DNA Gel Stain (Pishgam, Tehran, Iran).

The amplified fragments were sequenced by “Cardiogenetic Research Center” at “Shahid Rajaee Heart Hospital” (Tehran, Iran) using Sanger method. Sequences were compared with those deposited in National Center for Biotechnology Information (NCBI) and edited using BioEdit v. 7.0.0^57^. Alignments were performed in CLUSTAL-W^58^. Maximum likelihood phylogenetic trees were constructed using MEGA X^59^ based on the lowest value of the Bayesian Information Criterion (BIC). Bootstrap values were calculated based on 1000 replicates.

### Permission to collect native plant material

This research was based on the MSc research proposal of Mohammadreza Hajialigol. As the project proposal (No. 126764) was accepted by College of Agricultural Technology, University of Tehran, we obtained permission to collect walnut samples. So the use and study of the walnut plant was compiled with local and national regulations.

### Ethical approval

This research does not contain any studies with human participants or animals.

We confirm that all the methods were carried out in accordance with relevant Institutional guidelines and regulations.

## Data Availability

Housekeeping genes sequences are available from GenBank with accession numbers: 16S rRNA: OP048974, OP048975, OP048976, OP048977. *gyrB*: OP057241, OP057242, OP057243, OP057244, OP057245, OP057246, OP057247, OP057248, OP057249. *infB*: OP057250, OP057251, OP057252, OP057253, OP057254, OP057255, OP057256, OP057257, OP057258. *fusA*: OP057224, OP057225, OP057226. *leuS*: OP057227, OP057228, OP057229. *pyrG*: OP057230, OP057231, OP057232.
